# Patterns of DNA variation between the autosomes, the X chromosome and the Y chromosome in *Bos taurus* genome

**DOI:** 10.1038/s41598-020-70380-9

**Published:** 2020-08-12

**Authors:** Bartosz Czech, Bernt Guldbrandtsen, Joanna Szyda

**Affiliations:** 1 Biostatistics Group, Department of Genetics, Wroclaw University of Environmental and Life Sciences, Kozuchowska 7, 51-631 Wrocław, Poland; 2 Center for Quantitative Genetics and Genomics, Department of Molecular Biology and Genetics, Aarhus University, 8830 Tjele, Denmark; 3 Department of Animal Sciences, University of Bonn, Endenicher Allee 15, 53115 Bonn, Germany; 4 Institute of Animal Breeding, Krakowska 1, 32-083 Balice, Poland

**Keywords:** Computational biology and bioinformatics, Genomics, Evolutionary biology

## Abstract

The new ARS-UCD1.2 assembly of the bovine genome has considerable improvements over the previous assembly and thus more accurate identification of patterns of genetic variation can be achieved with it. We explored differences in genetic variation between autosomes, the X chromosome, and the Y chromosome. In particular, variant densities, annotations, lengths (only for InDels), nucleotide divergence, and Tajima’s D statistics between chromosomes were considered. Whole-genome DNA sequences of 217 individuals representing different cattle breeds were examined. The analysis included the alignment to the new reference genome and variant identification. 23,655,295 SNPs and 3,758,781 InDels were detected. In contrast to autosomes, both sex chromosomes had negative values of Tajima’s D and lower nucleotide divergence. That implies a correlation between nucleotide diversity and recombination rate, which is obviously reduced for sex chromosomes. Moreover, the accumulation of nonsynonymous mutations on the Y chromosome could be associated with loss of recombination. Also, the relatively lower effective population size for sex chromosomes leads to a lower expected density of variants.

## Introduction

DNA variation refers to differences in DNA sequence among individuals. Decreasing costs and reducing the time of whole-genome sequencing using the Next-Generation Sequencing (NGS) technology brings the opportunity to sequence many samples. The analysis of differences in DNA variation between autosomes and sex chromosomes plays an important role in understanding the evolution of chromosomes. In terms of studying variation, cattle is an interesting model, since for several generations it has undergone strong artificial selection toward increased milk (Holstein, Jersey, Jysk, Rd Dansk Malkerace anno 1970, Sortbroget Dansk Malkerace anno 1965) or beef (Danish Shorthorn) production. Besides, modern cattle is composed of breeds with distinct phenotypic characteristics. We used the new ARS-UCD 1.0.25 assembly that is the latest and the most accurate (less gaps) version of the cattle reference genome^1^. However, this assembly, like the UMD3.1^2^, does not contain the bovine Y chromosome (BTY), so most analyzes ignored the Y chromosome. As a result it has been omitted from most studies, so that in livestock, only one association was identified between genetic variants from BTY and phenotypes (in particular in pigs, source AnimalQTdb www.animalgenome.org/cgi-bin/QTLdb/index; Release 41). Recently the 1000 Bull Genomes Project^3^ added the Y chromosome sequence from the Btau 5.0.1 to the ARS-UCD, creating the ARS-UCD1.2_Btau5.0.1Y assembly. Thereby has become feasible to use all nuclear bovine chromosomes.

According to the ARS-UCD1.2_Btau5.0.1Y reference genome assembly, the bovine genome spans 2,759,153,975 bps with 30,278 genes (GeneBank assembly accession: GCA_002263795.2 and GCA_000003205.6) and consists of 29 autosomes and two sex chromosomes—X and Y (Table 1). The hemizygous Y chromosome in *Bos taurus* is short and contains only a few genes. In contrast, the bovine X chromosome (BTX) contains many more genes and its length is similar to the BTA2. Chromosome 1 is the longest bovine chromosome, spanning 158,534,110 bps with 1,218 genes, while chromosome 25 is the shortest bovine chromosome (949,746 bps shorter than BTY), spanning 42,350,435 bps with 1,611 genes. Regarding the ratio of the number of genes to the total contig length (chromosome), we can see that BTA25 has the highest ratio (38.04), while the Y chromosome has the lowest ratio (4.76). It is also worth to mention, that the karyogram of cattle chromosomes reports BTA29 as the shortest chromosome. BTY contains the lowest number of genes (206), while chromosome 23 contains the highest number of genes (1,708). Clearly, chromosome length is not linearly related to the number of genes. Moreover, 29 autosomes are acrocentric, while both sex chromosomes (X and Y) are submetacentric. Since recombination that generates new combinations of alleles is characteristic to autosomes, on sex chromosomes, this phenomenon is reduced in males to only small homologous regions shared between BTX and -Y, called the pseudoautosomal regions (PARs). BTY is poorly characterized because it is difficult to sequence due to the occurrence of a high proportion of repetitive sequences^4^.

Differences in genetic variation patterns arise from variable recombination and mutation rates, genetic drift, demography, selection, and population history. Therefore the focus of our study was on the comparison of patterns of genetic variation between autosomes, the X chromosome, and the Y chromosome in the context of the bovine genome.

**Table 1:** Summary of ARS-UCD1.2_Btau5.0.1Y reference genome.

Chromosome	Length of chromosome [bp]	Number of genes	Number of genes/Mbp ratio
1	158,534,110	1216	7.67
2	136,231,102	1284	9.43
3	121,005,158	1287	10.64
4	120,000,601	593	4.94
5	120,089,316	1080	8.99
6	117,806,340	692	5.87
7	110,682,743	1264	11.42
8	113,319,770	898	7.92
9	105,454,467	800	7.59
10	103,308,737	1554	15.04
11	106,982,474	1620	15.14
12	87,216,183	1194	13.69
13	83,472,345	479	5.74
14	82,403,003	864	10.49
15	85,007,780	738	8.68
16	81,013,979	1127	13.91
17	73,167,244	446	6.10
18	65,820,629	948	14.40
19	63,449,741	526	8.29
20	71,974,595	387	5.38
21	69,862,954	412	5.90
22	60,773,035	845	13.90
23	52,498,615	1708	32.53
24	62,317,253	1048	16.82
25	42,350,435	1611	38.04
26	51,992,305	849	16.33
27	45,612,108	1665	36.50
28	45,940,150	982	21.38
29	51,098,607	733	14.34
X	139,009,144	1222	8.79
Y	43,300,181	206	4.76

## Results

### Alignment to the reference genome

**Figure 1:**
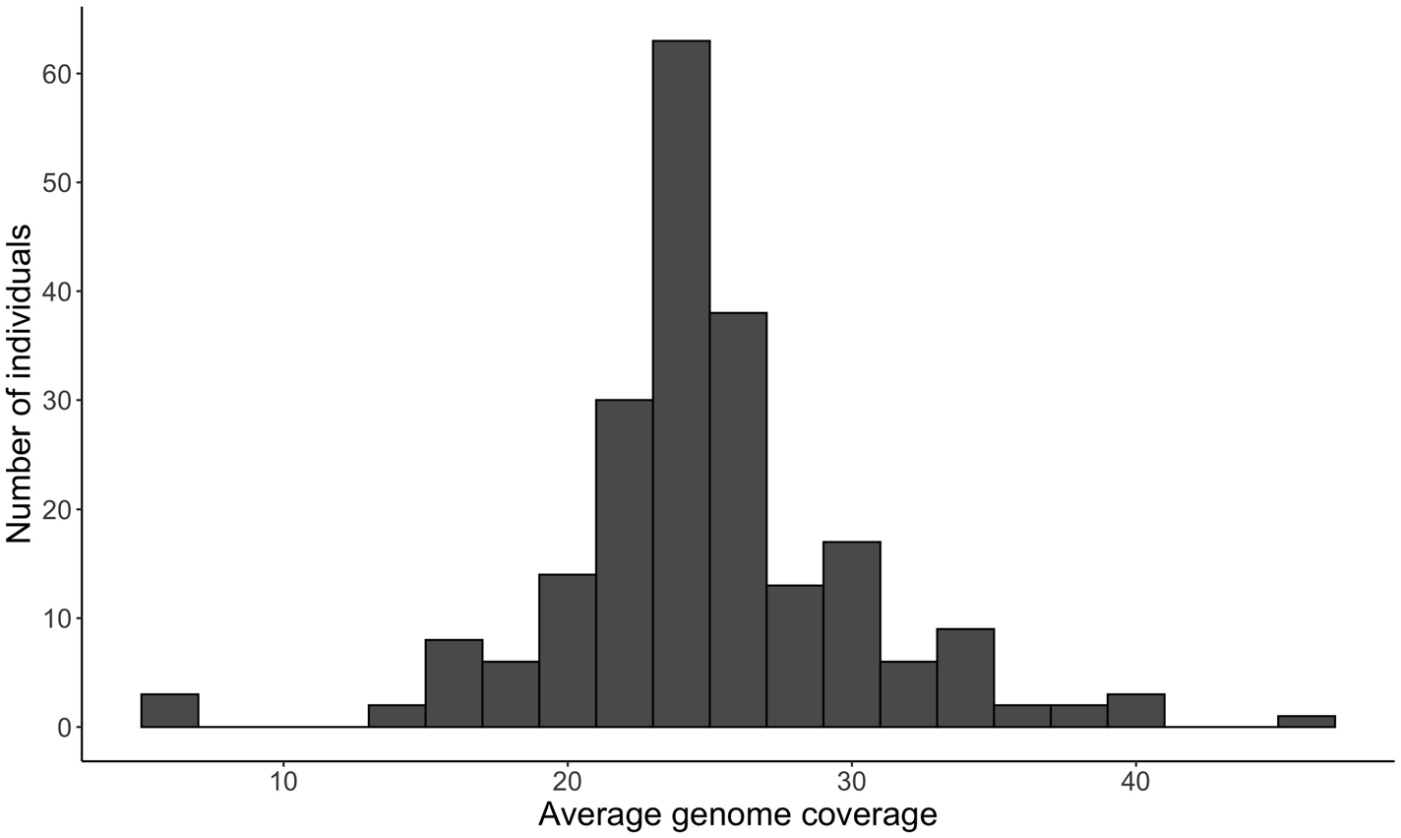
Average genome coverage.

The quality of alignment was expressed by the total percent of mapped reads and the percent of properly paired mapped reads (both reads are mapped close to each other in opposite directions on the same chromosome). The percent of mapped reads for each individual was very high and ranged from 91.4% to 99.9% with mean 99.7% ($\pm 0.6$) and very similar mode 99.9%. The percent of properly paired mapped reads was also high and varied between 89.3% and 99.1% with mean 97.1% ($\pm 1.7$) and mode 97.9%. Average genome coverage was calculated separately for each individual and ranged from 5.28 to 46.79 with mean and mode both equal to 25 (Fig. 1). Individuals average genome coverage less than 15, while two individuals have the average genome coverage above 40. The individual with the lowest average genome coverage (5.28) contains 99.6% of mapped reads and 97.8% of properly paired mapped reads. The individual with the highest average coverage (46.79) contain 99.9% of mapped reads and 97.9% of properly paired mapped reads.

### Variation

**Figure 2:**
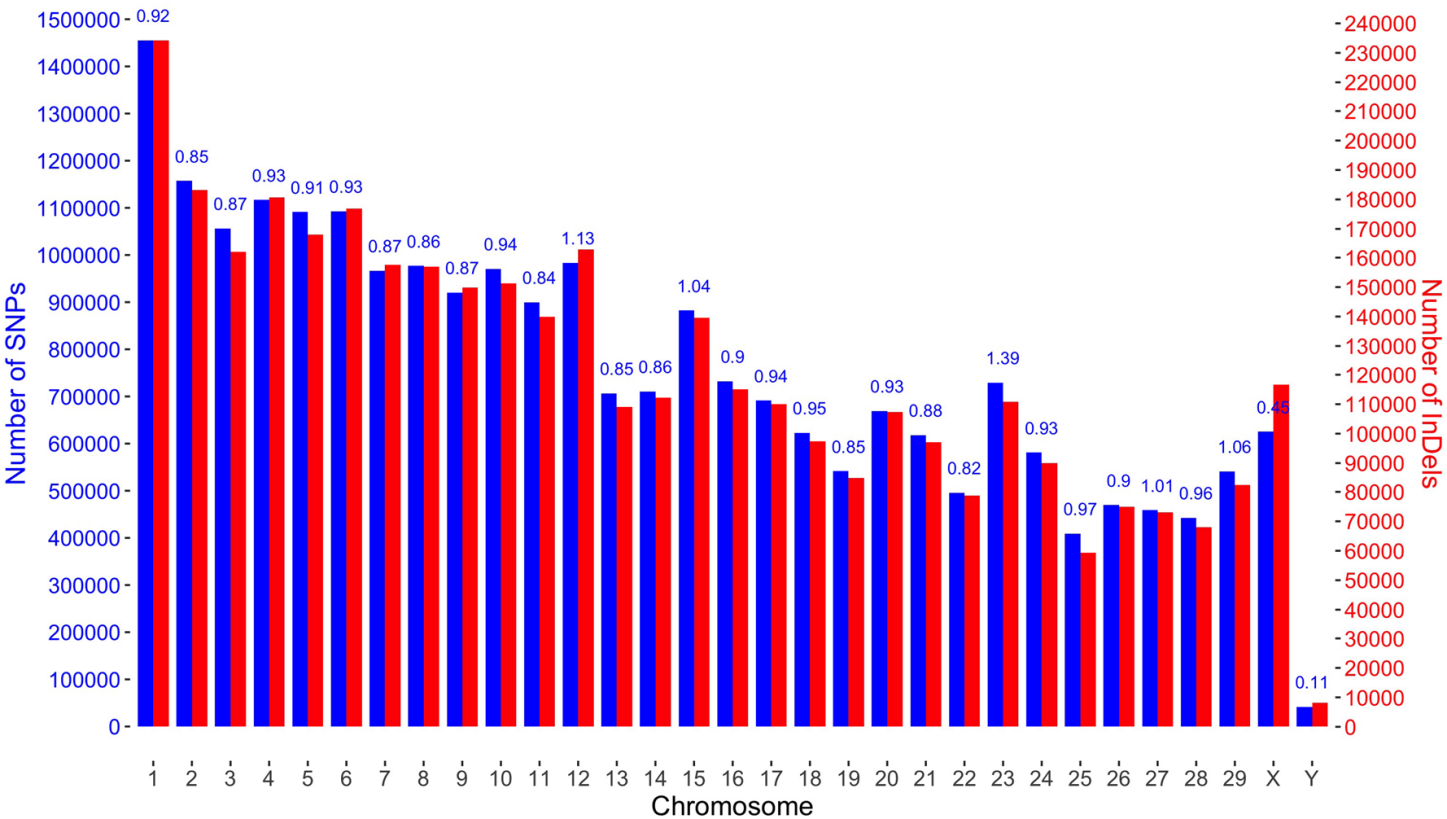
The distribution of variants. Blue bars with the y-axis on the left side represent SNPs, while red bars with the y-axis on the right side represent InDels. Values above bars represent the percent of the total length of chromosome covered by SNPs.

Overall, 27,414,076 variants were identified (Fig. 2). Of these, 86.3% were SNPs, and 13.7% were InDels. BTA1 contained the highest number of variants (1,689,556), while the BTY contained the lowest number of variants (49,591). The total number of SNPs was 23,655,295, 0.9% of the total genome length. 9,848,025 SNPs were located within coding sequences (CDS). BTA1 contained the highest number of SNPs (1,455,295 with 530,272 SNPs in coding regions), but the highest SNP density, expressed by the proportion of the number of SNPs to chromosome length, was highest for BTA25 (2.6%). BTY was characterized by the lowest number of SNPs (41,500; 4,185 in coding regions) and the lowest SNP density (0.1%). 3,758,781 of InDels were identified including 1,591,937 in coding regions. BTA1 had the highest number of InDels (234,261), whereas BTY had the lowest number of InDels (8,091) (Fig. 2).

**Figure 3:**
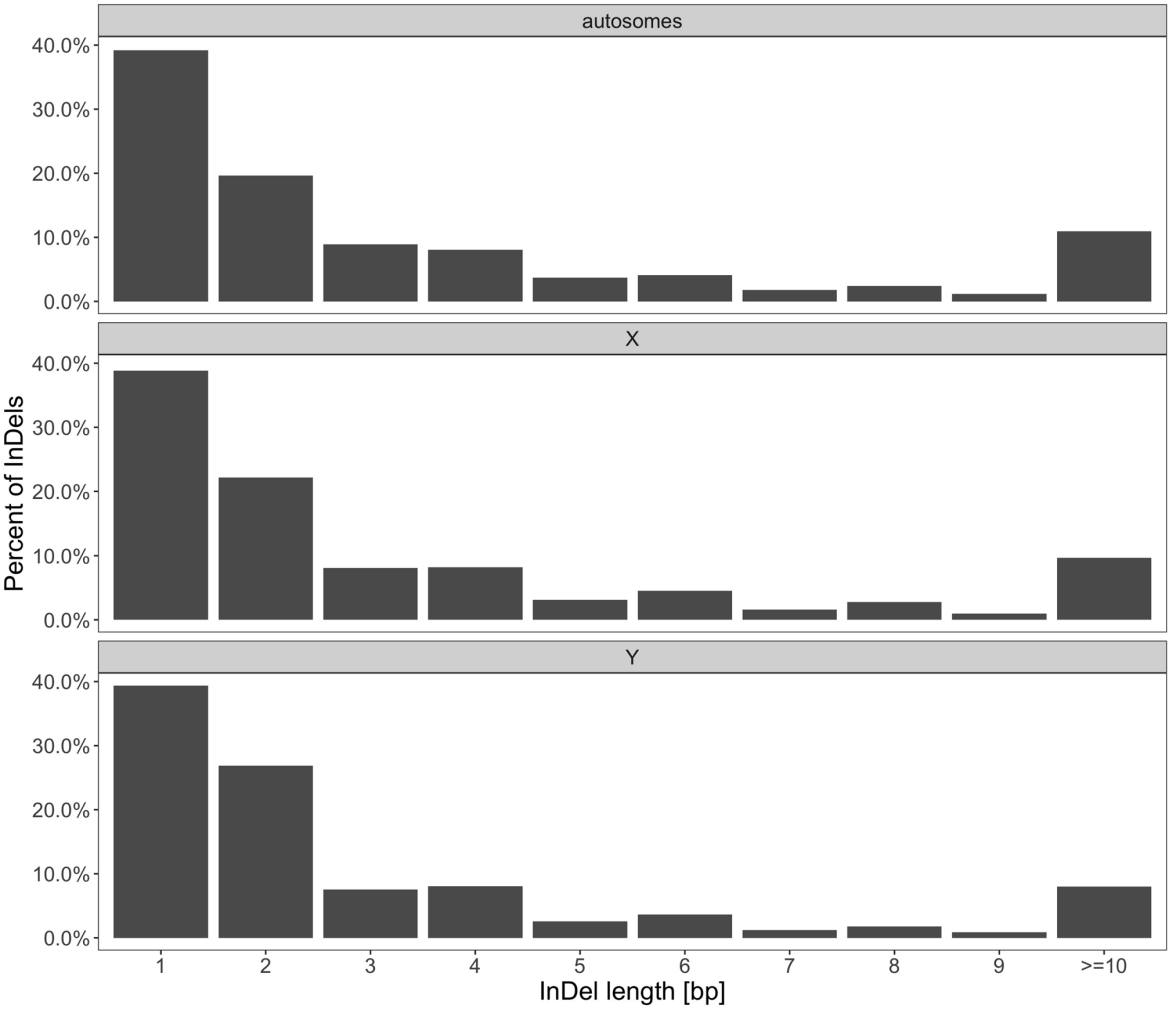
Length distribution of deletions and insertions for autosomes and sex chromosomes.

The length of InDels varied between 1 and 281 bps on autosomes, from 1 to 233 bps on BTAX, and from 1 to 156 bps on BTY and is not uniformly distributed ($P < 0.001$). The most frequently observed length of InDels was one bp, but the median length was two bps (Fig. 3). InDel median length differed significantly ($P < 0.001$) between autosomes and BTX as well as between autosomes and BTY. 12%, 19%, and 98% of indels located respectively on autosomes, BTX and, BTY, was longer or equal 10bps.

### Distribution of polymorphisms

**Figure 4:**
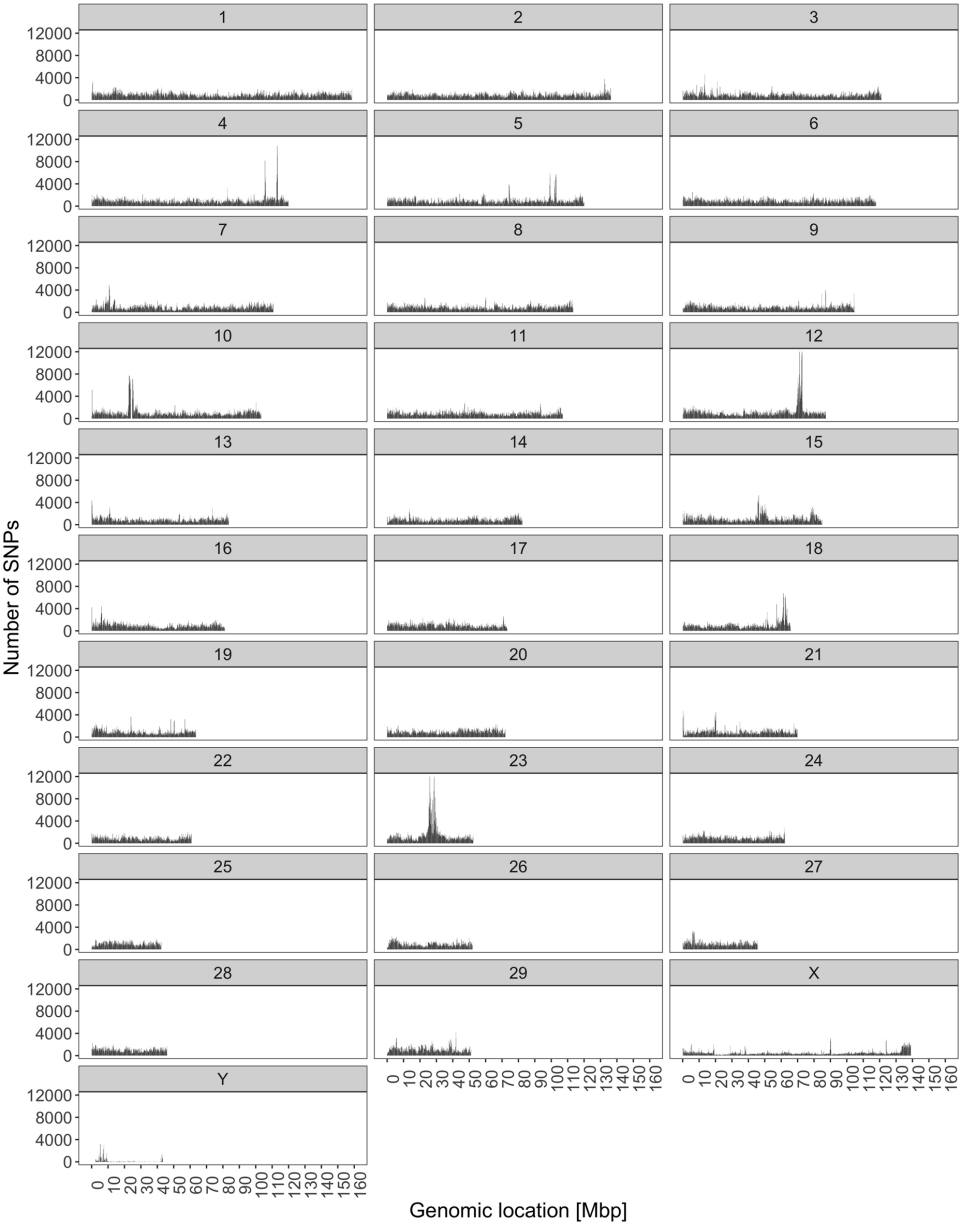
The distribution of SNPs across chromosomes.

The analysis of variant distribution was carried out by counting the number of variants within 100 kbp non-overlapping windows (Fig. 4). The number of SNPs per 100 kbp bin was heterogeneous across windows within the same chromosome ($P < 0.001$). The highest number of SNPs in one window (16,184) was observed on BTA12. On the other hand, on four chromosomes we identified windows without SNPs: BTA8 (one window), BTA9 (eight windows), BTA10 (eight windows), and BTY (58 windows). All of the windows were annotated as intergenic. However, 27 out of the total 58 windows on BTY corresponded to gaps in the reference genome marked by stretches of Ns. Conversely, windows with the number of SNPs exceeding 10,000 were found on BTA4 (one window with 10,838 SNPs) 8% of window length overlapped with genes, BTA12 (four windows with 10,431, 11,487, 15,097 and 16,186 SNPs) in 3% of windows length overlapped with genes, and BTA23 (three windows with 11,706; 12,452 and 13,229 SNPs) in which 100% of windows length overlapped transcription regions. Interestingly, the functional annotation showed that four out of the eight SNP-rich windows overlapped with lncRNA genes.

**Figure 5:**
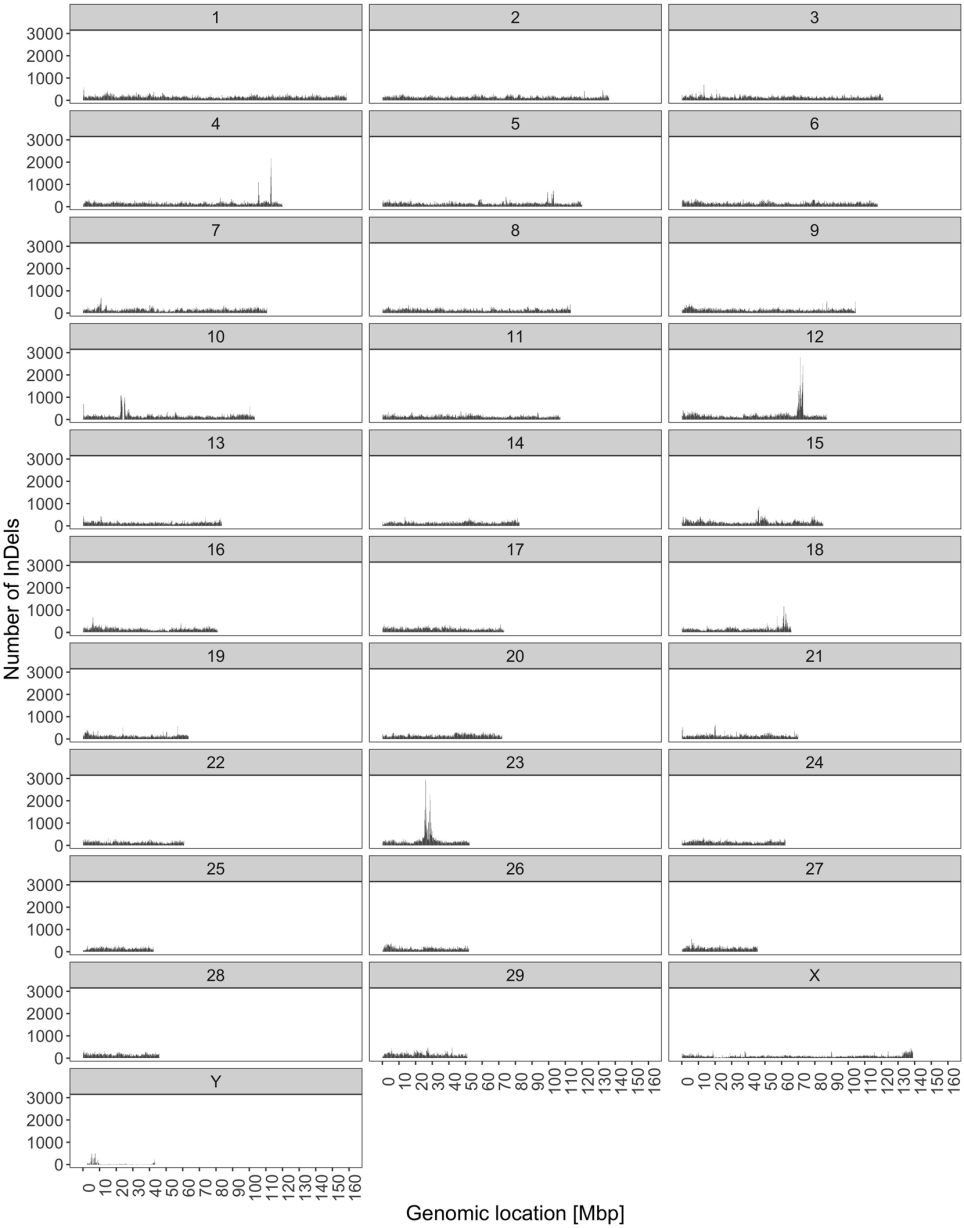
The distribution of InDels across chromosomes.

The highest overall number of InDels was identified on BTA23 (2,930 InDels). InDel density (Fig. 5) was not uniform across the genome ($P < 0.001$). We identified 122 windows without InDels—seven on BTA9, eight on BTA10, and 107 on BTY. All the windows on BTA7 and BTA8 were intergenic. On BTY, 64 of the windows were intergenic, whereas 35 windows corresponded to gaps in the reference assembly. However, eight windows intersected 10 genes. 1% to 13% of these windows overlapped with the sequence of a gene. Windows harboring more than 2,000 InDels were found on BTA4 (one window with 2,162 InDels), BTA12 (two windows with 2,405 and 2,792 InDels), and BTA23 (three windows with 2,022; 2,258, and 2,930 InDels). Each of these windows overlapped with genes comprising from 33% of a window length to 100% of length, both on BTA23. As for SNP-rich windows, three out of the six InDel-rich windows overlapped with lncRNA genes.

### Variant annotation

**Figure 6:**
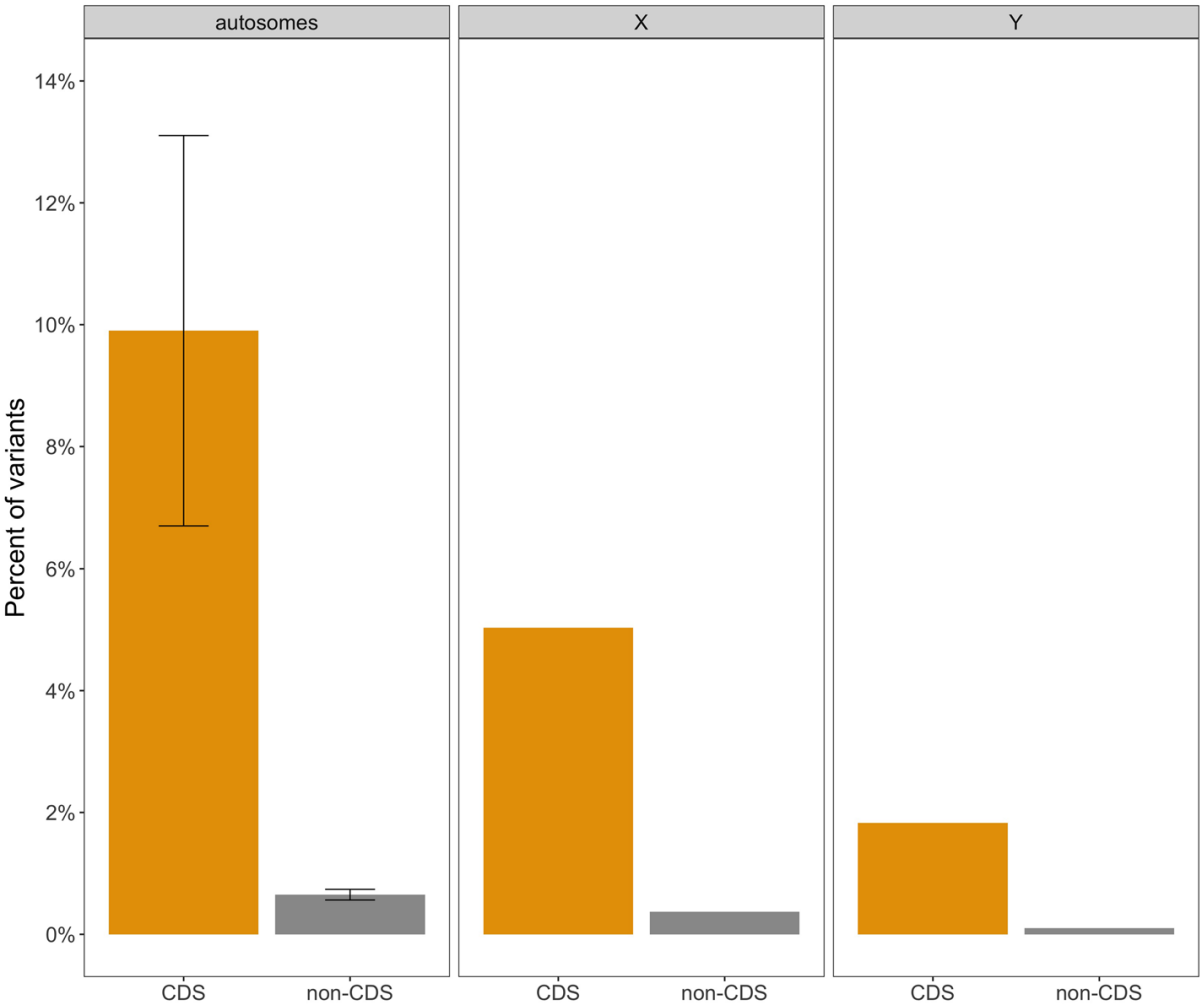
The annotation of variants in coding (CDS) and non-coding (non-CDS) regions.

The annotation of variants expressed as the percent of polymorphic bases located in non-coding and coding regions in relation to their length was summarized in Fig. 6. For each chromosome, the proportion of number of polymorphic sites to the length of the specific region (coding or non-coding) was higher for coding regions in comparison to non-coding regions. The proportion of the number of variants overlapping coding regions to the total length of this region was the highest on autosomes (9.90 ± 3.20), while the lowest on BTY (1.83). The proportion of the number of variants overlapping non-coding regions to the total length of this region was also observed on autosomes (0.65 ± 0.09) with the lowest proportion on BTY (0.10).

Focusing only on genes, on autosomes and BTX we observed the highest percent of variants was located in introns (41.8% ± 2.9% and 42.6%, respectively) and in non-coding transcripts (41.7% ± 2.4% and 41.7%, respectively). The Y chromosome showed the highest percent of variants in regions annotated up to 5,000 bps upstream of the 3’ end of gene start (25.3%) and up to 5,000 bps downstream of the 5’ end of gene end (24.8%). It was accompanied by a proportion of intronic and non-coding transcript variants being by 20% lower than in BTA and BTX. Moreover, variants with high (splice acceptor, splice donor, stop gained, frameshift variant, stop lost, start lost) and moderate (missense) impacts on proteins occurred more frequently on BTY than on BTA and BTX, while the latter had the lowest percent of variants with high or moderate impacts. Interestingly, on BTY 0.11% of variants in transcription regions were frameshift, which is much higher than on BTA (0.02%) and BTX (0.01%). The same tendency was observed for stop gained variants, which constituted 0.03% of BTY, but only 0.004% of BTA and 0.003% of BTX.

**Figure 7:**
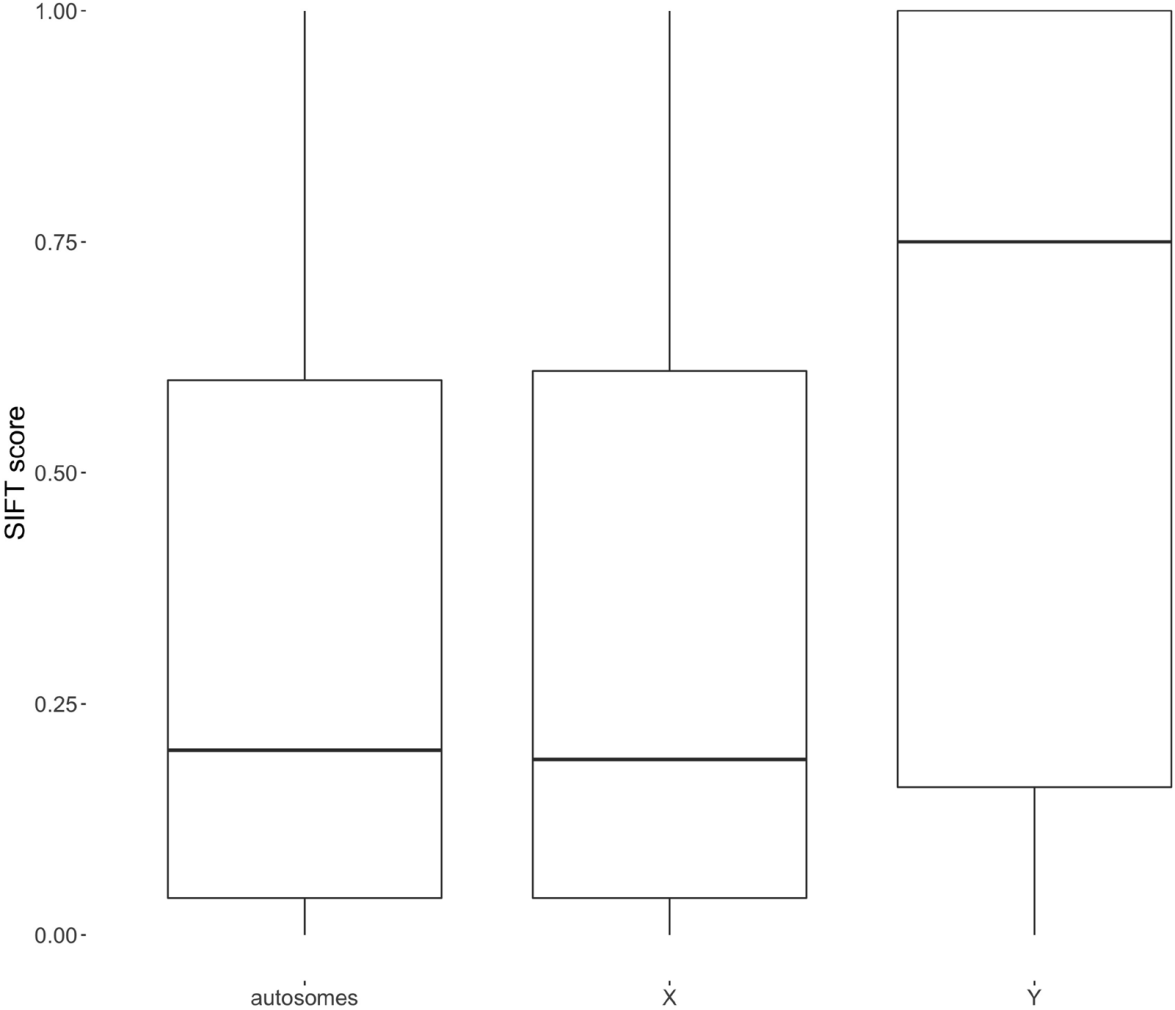
SIFT score for nonsynonymous and missense variants on autosomes, the BTX, and the BTY.

Nonsynonymous and missense variants were examined in order to predict whether an amino acid substitution affects protein function. For this purpose, the SIFT score was predicted. A variant with score 0 to 0.05 is considered deleterious while > 0.05 are considered tolerated. Figure  7 reports the distribution of SIFT scores among nonsynonymous and missense variants. We can see, that the distributions of SIFT scores for autosomes and the X chromosome are the same ($P = 0.76$), while differences between the X chromosome and the Y chromosomes as well as between the autosomes and the Y chromosomes are different ($P < 0.001$). The Y chromosome has the highest median of the SIFT score.

### Population genetics

**Figure 8:**
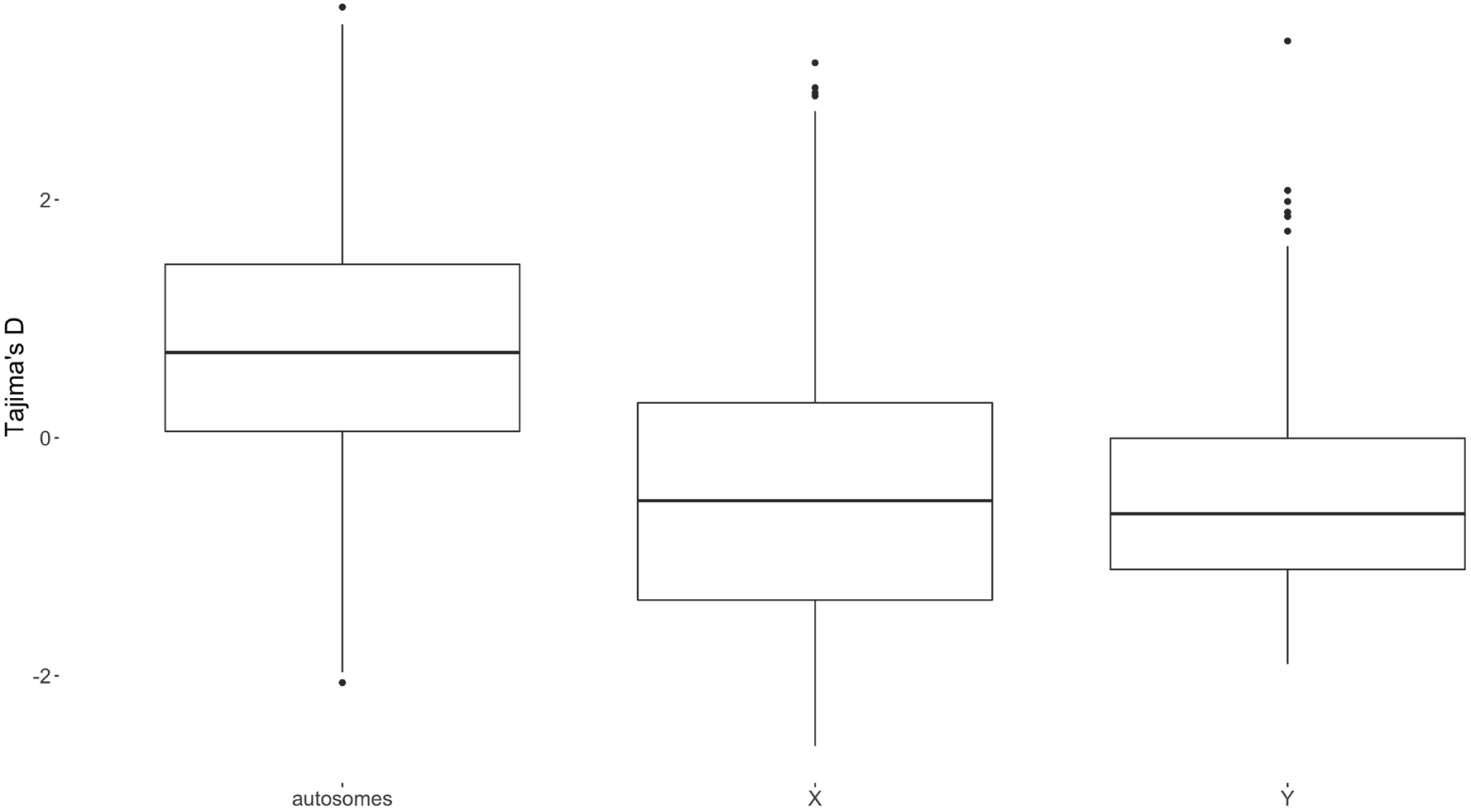
Tajima’s $D$ for autosomes, the BTX chromosome, and the BTY chromosome.

**Figure 9:**
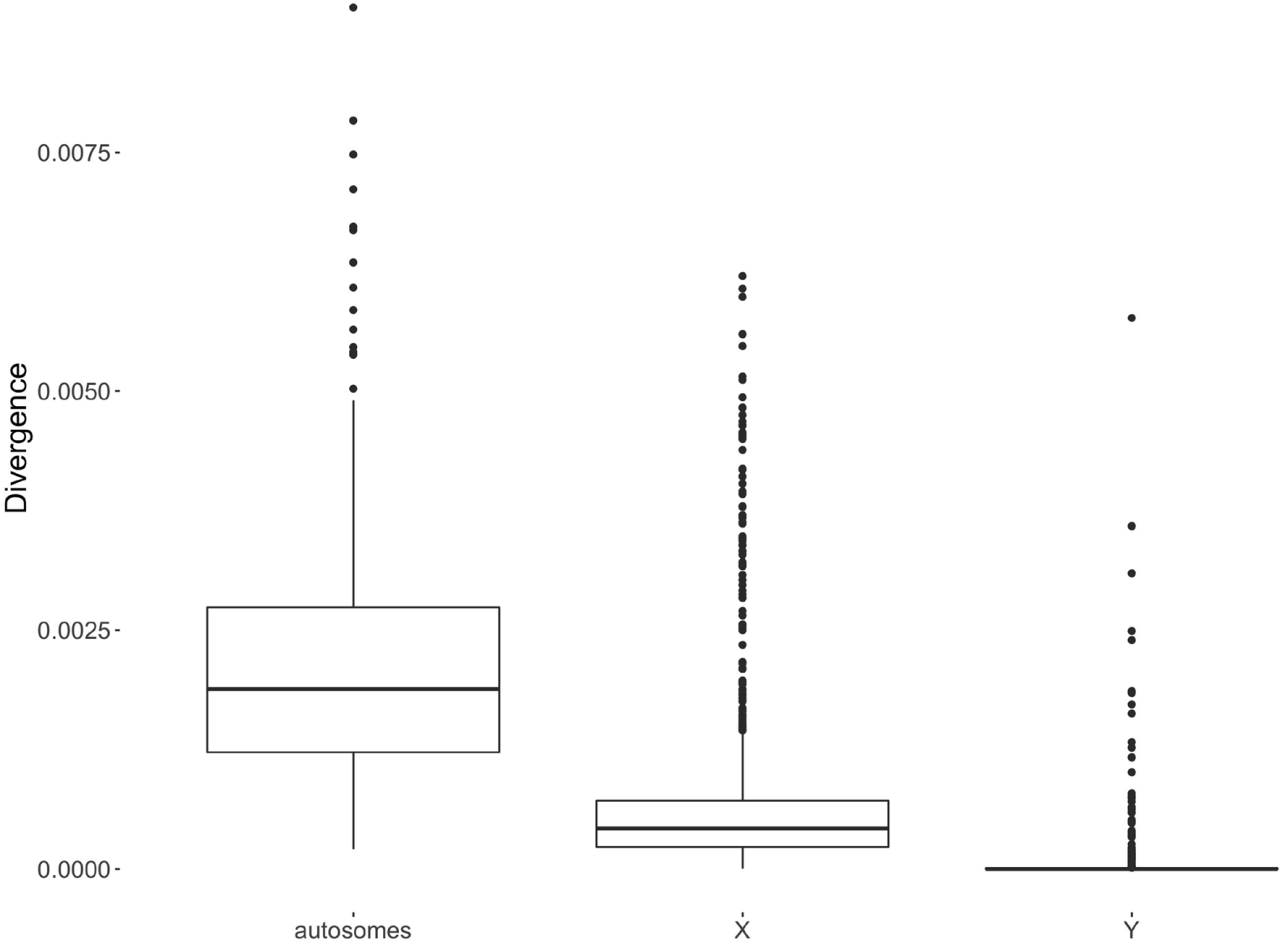
Nucleotide divergence for autosomes, the BTX chromosome, and the BTY chromosome.

The ratio of nonsynonymous to synonymous SNPs ($K_a/K_s$ ratio) was the highest for BTY (2.00) and the lowest for BTX (0.62). Autosome averaged ratio was also below unity (0.79 ± 0.15) and varied between 0.57 for BTA22 and 1.10 for BTA4, BTA15, BTA18, and BTA23. Tajima’s *D* statistic significantly differed between all three chromosome groups ($P < 0.001$) and was positive for autosomes and negative for both sex chromosomes (Fig. 8). Nucleotide diversity had the highest median for autosomes, followed by BTX and BTY (Fig. 9). All differences in distributions of nucleotide divergence between chromosome groups were significant ($P < 0.001$). Furthermore, windows with extremely high diversity and windows with extreme values of Tajima’s *D* statistic were examined for their SNP density. These windows do not overlap regions with high SNP density.

## Discussion

Our goal with this study was to compare patterns of genetic variation between autosomes, the BTX, and the BTY. A high percent of mapped reads, a high percent of properly mapped paired reads, and the fact that the most of individual genomes were sequenced with a genome average coverage exceeding twenty, demonstrated high quality of our data allowing for reliable inferences. Interestingly, individuals with low percent of mapped reads and low percent of properly mapped reads had high genome average coverage. This implies no correlation between genome average coverage and percent of mapped and properly mapped reads.

Altogether, over 23.6 million SNPs and over 3.7 million InDels were identified, among them 24% of SNPs and 94% of InDels were novel. Such proportion between novel SNPs and InDels can be caused by the fact that many more studies have been related to SNPs and thus the SNP database is more complete^5^. A larger number of SNPs than InDels is not an uncommon observation. InDels in transcription regions are highly deleterious to gene function as they can completely alter protein amino acid sequence by changing the open reading frame (frameshift mutation). BTY contains the lowest number of variants, which is not only due to its length. For instance, BTA25 is the shortest autosome, but it contains much higher number of variants. Taking into account the length of each chromosome, we observed that only 0.1% of the whole length of the BTY contained SNPs, where it is 0.45% for BTX. On the other hand, the shortest chromosome (BTA25) exhibited the highest SNP density of 2.58%. One of the factors affecting DNA variation is the effective population size ($N_{e}$). Estimated based on autosomes, the effective population size is higher than based on sex chromosomes—3/4 of autosomal $N_{e}$ for X and 1/4 of autosomal $N_{e}$ for Y^6^. The lower $N_{e}$, the more important role of genetic drift, so we expect that the effect of genetic drift is the highest for the Y chromosome, which furthermore implies lower neutral diversity of Y^7^. This observation contradicts the theory of male mutation bias that assumes that more mutations accumulate in male germline due to a greater number of male germline cell divisions, which is especially pronounced for BTY which “spends more time” in male germline than the other chromosomes^8^. A difference observed in our study is possibly due to gaps in the BTY chromosome assembly of the bovine genome (i.e. a high number of unknown nucleotides), which humper an accurate comparison of variant density between autosomes, BTX and BTY.

However, more recent studies emphasize that the comparison of mutation patterns on a general, chromosome-wide scale, is not valid due to a strong variation in local mutation rates^9,10^. Also in our study SNP and InDel distribution showed a non-random pattern, characterized by mutation hotspots with very high variant density, albeit only on autosomes. We observed that SNP density is positively correlated with indel density, which is in agreement with results obtained for humans by Hodgkinson et al.^11^. Estivill et al.^12^ showed that regions with high-density of SNPs are correlated with segmental duplications in the human genome. Varela and Amos^13^ declared that regions with unusually high recombination rates tend to have a high density of SNPs, while Aggarwala and Voight^14^ estimated the effect of DNA sequence k-mers on SNP probability.

Based on the annotation of transcription regions, it can be shown that there is a tendency that variants in transcription regions on BTX have less severe consequences as compared to Y and autosomes. Fewer extreme variants are consistent with purging due to the hemizygous state in males. On autosomes, these would most of the time be hidden in males due to diploidy. Based on Ka/Ks ratio, we also observed that on the BTY there is a tendency to accumulate more nonsynonymous than synonymous substitutions (Ka/Ks = 2.00). In contrast, BTX shows a much lower ratio (Ka/Ks = 0.62). Such differences might arise from the tendency for the degeneration of the Y chromosome—Haldane’s rule, which indicates faster male evolution and reason of accumulation of nonsynonymous variants, mainly due to the lack of the recombination^15^. In males, BTX is hemizygous, so any, even slightly deleterious mutation has an effect on the phenotype. For mammalian genomes such situation was demonstrated in a simulation study by Mackiewicz *et al*.^16^

## Methods

### Material

The material comprised whole-genome DNA sequences of 217 individuals representing Holstein (69 bulls and 6 cows), Jersey (41 bulls), Jysk (5 bulls), Rd Dansk Malkerace (46 bulls), Rd Dansk Malkerace anno 1970 (15 bulls and 5 cows), Sortbroget Dansk Malkerace anno 1965 (15 bulls and 5 cows) and Danish Shorthorn (5 bulls and 5 cows) breeds available courtesy of the Center for Quantitative Genetics and Genomics at the Aarhus University. Animals were not closely related. There can be found some distant relationship within the breeds.

Whole-genome DNA sequences were obtained by the Illumina HiSeq 2000 Next Generation Sequencing platform and similar short-read sequencing platforms. Access to this data was available via the computer cluster of the Center for Quantitative Genetics and Genomics at Aarhus University. ARS-UCD1.2_Btau5.0.1Y^1^ reference genome was used to processing whole-genome sequence data. This genome represents the latest reference genome of *Bos taurus* additionally assembled with the Y chromosome (Btau5.0.1) from Baylor College^17^. In this study two GFF (General Feature Format) files were used for the annotation of identified variants. Btau_5.0.1 and ARS-UCD1.2 GFF were merged to obtain a complete annotation file for each chromosome. Those files were downloaded from the NCBI database (ftp://ftp.ncbi.nlm.nih.gov/genomes/all/GCF/002/263/795/GCF_002263795.1_ARS-UCD1.2/ and ftp://ftp.ncbi.nlm.nih.gov/genomes/all/GCF/000/003/205/GCF_000003205.7_Btau_5.0.1/). The actual assembly file used was downloaded from https://sites.ualberta.ca/~stothard/1000_bull_genomes/ARS-UCD1.2_Btau5.0.1Y.fa.gz.

### Processing the next-generation sequencing data

The FastQC software was used to summarize and visualize sequence quality^18^. Sequence quality metrics measure the probability that a given base is called incorrectly. Low quality bases were trimmed from reads using Trimmomatic^19^ with options SLIDINGWINDOW:3:15, LEADING:3, TRAILING:3, and a minimum read length of 70 bp. Cleaned reads were aligned to the reference genome by using the Burrows-Wheeler Aligner (BWA) with the MEM algorithm^20^. After alignment, the flagstat tool in SAMtools^21^ was used to calculate the percent reads correctly mapped to the reference genome. The genome average coverage, representing the number of times that a base in the reference genome was covered by aligned reads^22^, was calculated for each individual, using the genomecov tool in bedtools^23^. It was expressed by coverage$=\frac{R \cdot L}{G}$, where *R* is the total number of aligned reads, *L* is average read length and *G* is the genome size. The output from BWA-MEM was piped to SAMtools^21^ and then compressed to the BAM format (Binary Alignment Map)—a binary version of the SAM format. Afterwards, SAMtools fixmate^21^ was run to adjust the mate-read position. BAM files were sorted using SAMtools^21^. PCR duplicates were marked using MarkDuplicates from Picard^24^. Finally, Base quality scores were recalibrated using Genome Analysis Toolkit (GATK)^25^.

### Variant calling

Variant calling allows the identification of differences between analyzed sequences and the reference genome, i.e., of polymorphic sites. First, GATK’s HaplotypeCaller^25^ was used to create files (gVCF files) that summarize information on sites potentially deviating from the reference. Specifically, the tool identifies genomic regions, so-called ActiveRegions, which contain significant differences from the reference genome. Those regions are then processed by HaplotypeCaller. Other variations (non ActiveRegions) are skipped in order to accelerate the analysis. Afterward, ActiveRegions are used to construct haplotypes by building a De Bruijn-like graph^26^ and calculate haplotype frequencies. Haplotypes were realigned against the reference haplotype using the Smith-Waterman algorithm^27^ to identify potentially polymorphic sites. Then a matrix of likelihoods of haplotypes given the DNA sequence of reads was calculated using Hidden Markov Models. Thereafter, HaplotypeCaller assigned the most likely genotypes^25^. The standard phred-scaled confidence threshold of 30 was used to remove the potential false-positive variants. The last step of variant calling comprised merging gVCF files representing different individuals. For this purpose, GATK GenotypeGVCFs was used^25^. This step resulted in raw variant call files (VCF), which contained summary information of each detected variant.

After variant calling, the identified polymorphisms were annotated with predicted biological consequences and functions. For each polymorphic variant the associated Sequence Ontology (SO)^28^ terms, which categorize genomic functions of the coding sequence, were assigned. For this purpose, the snpEff software was used^29^. The program uses information included in the annotation file (GFF) to assign an annotation to each detected variant. In addition, we predicted effects of nonsynonymous and missense variants on protein functions using the SIFT score. For prediction of effect of amino acid substitution we used SIFT4G software^30^. Variants with SIFT score $\le 0.05$ were classified as deleterious, while $> 0.05$ were considered tolerated^31^.

### Statistical analysis

#### The Shapiro–Wilk test

The Shapiro–Wilk^32^ was used to test whether a sample was obtained from a normal distribution, comparing the following hypotheses:

$$H_{0}:$$
The distributions of Tajima’s D and nucleotide divergence follow the normal distribution
$$H_{1}:$$
The distributions of Tajima’s D and nucleotide divergence do not follow the normal distribution

The corresponding test statistic is given by:

$$\begin{aligned} W=\frac{(\sum _{i=1}^{n} a_{i}x_{(i)})^{2}}{\sum _{i=1}^{n}(x_{i}-\bar{x})^2} , \end{aligned}$$

where $a_{i}$ is the tabulated Shapiro–Wilk coefficient, $x_{(i)}$ is the *i*th smallest value of the Tajima’s D/nucleotide divergence and $\bar{x}$ is the mean of Tajima’s D/nucleotide divergence. We reject $H_{0}$ at the significance level ($\alpha = 0.05$) if $W<W_{\alpha }$, where $W_{\alpha }$ is tabulated critical threshold for Shapiro–Wilk.

#### The $\chi ^{2}$ goodness-of-fit test

The $\chi ^{2}$ goodness-of-fit test was used to check whether the observed variable in the general population follows the uniform distribution. Corresponding hypotheses were defined as follows^33^:

$$H_{0}:$$
The distribution of the observed variable is uniform
$$H_{1}:$$
The distribution of the observed variable is not uniform

The $\chi ^{2}$ goodness-of-fit statistic is given by^34^:

$$\begin{aligned} \chi ^{2}= \sum \limits _{i=1}^{k} \frac{(O_{i}-E_{i})^{2}}{E_{i}}, \end{aligned}$$

where $O_{i}$ is the observed count of observations in the *i*th group, $E_{i}$ is the count of observations expected under the uniform distribution, and *k* is the number of groups. Under $H_{0}$, the test follows the $\chi ^{2}$ distribution with $k-1$ degrees of freedom.

#### The Kruskal–Wallis test

The Kruskal–Wallis test is a non-parametric equivalent of the *F* test in the analysis of variance for non-normal data. Corresponding hypotheses are^35^:

$$H_{0}:$$
There is no difference among *k* populations’ median
$$H_{1}:$$
At least one population’s median differs from the median of the other populations

The Kruskal–Wallis statistic is given by^36^:

$$\begin{aligned} H=\frac{12}{N(N+1)}\sum  \limits _{i=1}^{k} \frac{R_{i}^{2}}{n_{i}}-3(N+1) , \end{aligned}$$

where *N* is the total sample size, *k* is the number of groups, $R_{i}$ is the sum of ranks in the *i*th group, and $n_{i}$ is the size of the *i*th group. Under $H_{0}$, the distribution of this test can be approximmated by the $\chi ^{2}$ distribution with $k-1$ degrees of freedom.

#### The Mann-Whitney U test

The Mann-Whitney *U* test is the non-parametric equivalent of the *t*-test for two independent samples with the corresponding hypotheses^37^:

$$H_{0}$$
There is no difference between the two populations’ medians
$$H_{1}$$
There is a difference between the two populations’ medians

The statistic is given by^38,39^:

1 $$\begin{aligned} U=MN+\frac{N(N+1)}{2}-\sum _{i=1}^{N}R(X_{i}) , \end{aligned}$$

where *n* is the size of the first group, *m* is the size of the second group, and $R(X_{i})$ is the rank assigned to the first group (Wilcoxon statistic). We reject the null hypothesis $H_{0}$ at the significance level $\alpha$ if $t_{n,m,1-\frac{\alpha }{2}}<U<t_{n,m,\frac{\alpha }{2}}$, where $t_{n,m,1-\frac{\alpha }{2}}$ and $t_{n,m,\frac{\alpha }{2}}$ are quantiles of the Mann-Whitney distribution. For large sample sizes, $U \sim \mathcal {N}(\mu _{U},\,\sigma _{U}^{2})$, where $\mu _{U}=\frac{mn}{2}$, $\sigma _{U}^{2}=\frac{mn(N+1)}{12}$, where $N=n+m$. This test was applied to test multiple, simultaneous hypotheses; therefore the Bonferroni correction was used to account for multiple testing^40^.

### Genetic statistics

#### Nucleotide divergence

One of the statistic most widely used to measure the degree of polymorphism in a chromosome is a nucleotide divergence ($\pi$). It is a measure proposed by Nei and Li^41^. $\pi$ quantifies the nucleotide diversity among several sequences. In our case, we estimated $\pi$ along each chromosome in 100 kb non-overlapping windows. The estimator $\hat{\pi }$ of nucleotide divergence calculated based on information from VCF files is defined as:

$$\begin{aligned} \hat{\pi }=\frac{\sum _{i=1}^{k}\left[ AN_{i}(AN_{i}-1)\right] +(w-k)p}{2\sum _{i=1}^{k}\left[ AC_{i}(AN_{i}-AC_{i})\right] } , \end{aligned}$$

where *k* is the number of variants within a given 100 kb window, $AN_{i}$ is the total number of alleles in called genotypes of *i*th variant, *w* denotes the window size [100 kbs], *p* is the number of pairwise windows comparisons, and $AC_{i}$ is the allele count in genotypes of *i*th variant (for each alternative allele).

#### Tajima’s D

Another statistic used in this study is Tajima’s *D*. It allows for the detection of the evidence of selection. Tajima^42^ proposed this statistic as a measure of a rate of a random (neutral) evolution of DNA sequence. In this study, Tajima’s *D* was estimated over a 100 kbs non-overlapping windows as follows:

$$\begin{aligned} \hat{D}=\frac{\hat{\theta }_{\pi }-\hat{\theta }_{W}}{\sqrt{{{\,\mathrm{Var}\,}}(\hat{\theta }_{\pi }-\hat{\theta }_{W})}} , \end{aligned}$$

where $\hat{\theta }_{\pi }$ is the average number of pairwise differences given by $\hat{\theta }_{\pi } = \frac{\sum \nolimits _{i<j}d_{i,j}}{\frac{n(n-1)}{2}}$. Here, $d_{i,j}$ represents the number of differences between individual *i* and *j*, $n={N\atopwithdelims ()2}$ is the number of pairwise sequences comparisons with *N* being the number of individuals. $\hat{\theta }_{W}$ is Watterson’s estimator of the expected number of segregating sites under neutrality ($\hat{\theta }_{W} = \frac{S}{\sum\nolimits _{i=1}^{n-1}(\frac{1}{i})}$, where *S* is the number of sites that segregate in the sample).

$D>0$ indicates population reduction or balancing selection, $D=0$ indicates neutral mutations, and $D<0$ indicates population expansion or purifying and positive selection^43^. The VCFtools^44^ was used to calculate the Tajima’s D statistic.

#### ${K_a/K_s}$ ratio

The $K_a/K_s$ ratio was used as a measure of the selection pressure. Based on this ratio we compared natural selection pressure on proteins between chromosomes^45^. $K_a$ represents the ratio of the number of nonsynonymous substitutions per nonsynonymous site, while $K_s$ is the number of synonymous substitutions per synonymous site. $K_a/K_s$ equal to one indicates a neutral selection, the positive ratio is equivalent to positive selection, while the negative ratio indicates purifying selection. The output of the SnpEff^29^ software was used to calculate $K_a/K_s$ ratio.

### Computing environment

All programs and scripts were written in the bash command language and executed on the Genomics High Performance Cluster (GHPC) at the Center for Quantitative Genetics and Genomics at Aarhus University. The computing unit was the Red Hat Enterprise Linux Client release 4.8.5-28 (Centos) with 250 GiB/node of memory and 6 Intel Core Processor CPUs. All statistical analyzes were done using R (version 5.1)^46^ in RStudio^47^ and visualized using the ggplot2 R package^48^.

## Data Availability

The subset of the whole-genome data used in this study is available at SRP039339 under PRJNA238491. For the remaining data, the Board of the 1000 Bull Genome Project Consortium should be contacted. Whole-genome sequences from Aarhus University are available only upon agreement with the breeding organization and should be requested directly from the authors.
